# Life after COVID-19: a better normal?

**DOI:** 10.1177/1757913920951591

**Published:** 2020-08-23

**Authors:** A Arora

**Affiliations:** School of Clinical Medicine, University of Cambridge, Cambridge CB2 0QQ, UK

*Anmol Arora looks at the positive changes within healthcare systems, the community and public health efforts seen as a result of the Covid-19 pandemic. This article reflects on behavioural change theories to discuss how changes can be maintained and to offer an insight into what a future after COVID-19 may look like.*

In the 1940s, Kurt Lewin, often referred to as ‘the father of social psychology’, proposed a model for understanding behavioural change within organisations, which has since become a key theory in the study of behaviour. His ‘Unfreeze-Change-Refreeze’ model formed part of an integrated approach to analyse change at group, organisational and even societal levels.^1^ Lewin was especially keen that his work be used to bring about societal change, which could resolve forms of social conflict.^1^ The model consists of three stages:

1. *Unfreeze.* Create dissatisfaction with the status quo by explaining what is wrong with the present situation.^2^ This creates an understanding that in order to survive and prosper the status quo must change.
2. *Change.* The leader(s) offer a solution, defining a new status quo and a vision for a new normality.
3. *Refreeze.* The changes must be cemented to prevent regressing backwards. This may involve redesigning reward systems and incentives for adhering to novel processes.

The COVID-19 pandemic has elicited a clear sense of dissatisfaction with the status quo, as evidenced by the fact that society has experienced transformational change in a very narrow time frame. From a public health perspective, the dissatisfaction with the status quo appeared quickly at the onset of the pandemic, with an immediate recognition that vaccination efforts and drug trials must be expedited.^3^ From a medical perspective, the dissatisfaction with the status quo facilitated a rapid rise in telehealth and increased collaboration between primary and secondary care.^4^ From a societal perspective, the dissatisfaction with the status quo was based on universal understanding that remote working would reduce spread of infection and that community efforts were needed to help those in need.^5^ While the pandemic has caused disruption to lifestyles, it has also brought about changes such as, reduced environmental impacts, revitalised community spirit and enhanced focus on funding for healthcare systems, which we should now aim to sustain.

Public health principles and research are garnering widespread media attention as we begin to see issues of vaccination, controlled trials, infectious diseases and protective behaviours battling for front pages in newspapers. A plethora of behavioural changes to improve outcomes in patients infected with severe acute respiratory syndrome coronavirus 2 (SARS-CoV-2) have emerged, with varying evidence bases. These suggestions range from decreasing alcohol intake to increasing vitamin D intake.^6,7^ If the behavioural changes from this pandemic can be ‘refrozen’, there is potential to accelerate years of public health–directed change within the space of a few months.

Nadler and Tushman^8^ describe two dimensions with which change can be analysed: whether it is reactive or anticipatory, and whether it is incremental or transformational. The scope for change available through the pandemic is both reactive and transformational, which leads to what Nadier and Tushman describe as a ‘recreation’. This change is rare and involves a response to a crisis which threatens the status quo unless radical deviations from that status quo are quickly made. These changes are substantial in size and scope, but they are also difficult to maintain since they must be enacted quickly with little room for error.

This recreational change fits with what is referred to as the ‘punctuated model of equilibrium’, a framework for political change in the context of complex social systems. The framework, as described by Baumgartner and Jones,^9^ posits that policymaking consists of long periods of stability, punctuated by sporadic periods of intense and rapid change. The revolutionary periods that punctuate the model act as an opportunity to disrupt pre-existing activities and replace them with a new equilibrium.^10^ On that basis, it could be argued that the COVID-19 pandemic may act as a punctuation mark for this model and an opportunity for change.

We can conceptualise societal behaviour as a constantly evolving entity, moving slowly in no defined direction. Occasionally, perhaps only once in a generation, there is an event which may cause a seismic change and redirect its course. Subscribers to Lewin’s theory of change may accept that we are currently in the early stages of a behavioural change process. The ongoing COVID-19 pandemic has continued to inflict strain on healthcare providers, while also bringing political bodies under unprecedented scrutiny. In some areas, there is clear dissatisfaction with existing systems as healthcare, politics, social behaviours and public health itself are all facing unprecedented scrutiny. These necessitate change and some degree of effort to maintain that change if it is to continue into a new equilibrium. With societal structures facing extreme tension, our attention must eventually shift towards how we can build and maintain an idealistic post-COVID-19 society once that tension is released.
